# From Dormant Collections to Repositories for the Study of Habitat Changes: The Importance of Herbaria in Modern Life Sciences

**DOI:** 10.3390/life13122310

**Published:** 2023-12-08

**Authors:** Mauro Mandrioli

**Affiliations:** Department of Life Sciences, University of Modena and Reggio Emilia, Via Campi 213/D, 41125 Modena, Italy; mauro.mandrioli@unimore.it

**Keywords:** herbaria, climate changes, molecular analyses, digitization, natural collections

## Abstract

In recent decades, the advent of new technologies for massive and automatized digitization, together with the availability of new methods for DNA sequencing, strongly increased the interest and relevance of herbarium collections for the study of plant biodiversity and evolution. These new approaches prompted new projects aimed at the creation of a large dataset of molecular and phenological data. This review discusses new challenges and opportunities for herbaria in the context of the numerous national projects that are currently ongoing, prompting the study of herbarium specimens for the understanding of biodiversity loss and habitat shifts as a consequence of climate changes and habitat destruction due to human activities. With regard to this, the *National Biodiversity Future Center* (active in Italy since 2022) started a large-scale digitization project of the *Herbarium Centrale Italicum* in Florence (Italy), which is the most important Italian botanical collection, consisting of more than 4 million samples at present.

## 1. From Figurative Herbaria to Phenological Studies

In recent decades, an increasing number of published papers reported a worldwide reduction in plant biodiversity as a consequence of climate change and habitat destruction due to human activities (such as agricultural expansion, urbanization, and mining) [1,2,3,4]. In particular, even if climate changes affect all pillars of biodiversity, particular concerns are related to “tipping points”, where the exceedance of ecological thresholds will possibly lead to irreversible shifts in ecosystems [1,2,3,4]. In this context, climate changes and anthropogenic pressure are at the basis of a very rapid and detrimental ecological shift in numerous habitats, and many plant species seem to be unable to adapt to the present conditions; thus, they are threatened by extinction [5,6]. As a consequence, the global plant extinction rate is currently 500 times higher than before the Anthropocene, and forty-five percent of the world’s known flowering plants could be threatened by extinction [7]. Since plants play fundamental roles in ecosystems, their loss will be detrimental to the health of ecosystems and to the services they provide to humans and other organisms; therefore, estimations of the threat status of plants should be a critical component in current life sciences [1,2,3,4,5,6,7].

In this context, the 3500 herbaria that are available worldwide preserve a precious time-stamped tangible record of botanical biodiversity that, at present, consists of more than 400 million specimens (according to a survey of The New York Botanical Garden, https://sweetgum.nybg.org/science/ih/, accessed on 30 October 2023). These collections not only represent a snapshot of both present and past plant biodiversity [8,9], but they also preserve different types of samples (from dried plants and seeds to fungi and algae) collected at different phenological stages. Moreover, several institutions also possess plant materials conserved in liquid solutions that can be used for different molecular analyses [10].

**Figure 1:**
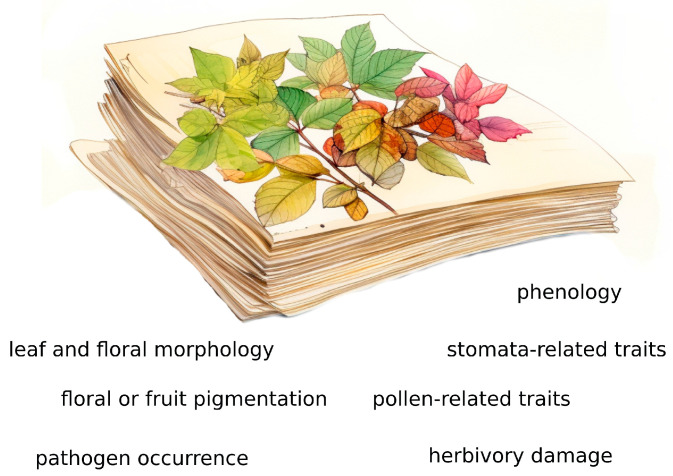
Herbarium specimens are sources of a large amount of data not only related to taxonomy but also to plant phenology and biology.

At the same time, even if herbaria include biases and errors in taxonomic, temporal, and spatial data, each herbarium specimen is accompanied by a large amount of metadata, including details about collection sites and dates, together with observations on phenology and notes made by collectors that may increase interest in such collections (Figure 1). For instance, plant collectors have long focused their attention on the reproductive parts (e.g., flowers and/or fruits) of sampled specimens, since they were critical to distinguishing species, but these traits may also have phenological significance, making herbaria enormous unintentional repositories of data about plant phenology [11].

The interest and relevance of herbaria have greatly increased in recent years. Their massive digitization not only made their composition/consistency available but also allowed for the downloading and analysis of high-resolution images related to each herbarium specimen, facilitating collaborative research on plant taxonomy since type specimens were also provided [12,13,14,15]. The availability of digitized copies of well-curated herbarium collections may therefore be useful to validate data in other herbaria since digital data can be used to correct species misidentification or to update species names according to new taxonomic insights [12,13,14,15].

At the same time, resources previously used for traveling to collections could instead be spent mining data simultaneously across many collections, via online open-access consolidated data portals, using digitized collections to identify and annotate specimens in other herbaria [10,14,16]. Interestingly, several digitization projects also made old and ancient figurative herbaria available, which reported data about the discovery, description, and distribution of each painted plant species; therefore, the availability of data on plant biodiversity from the 16th century actually indicates the anticipation of the advent of modern herbaria [17,18].

**Figure 2:**
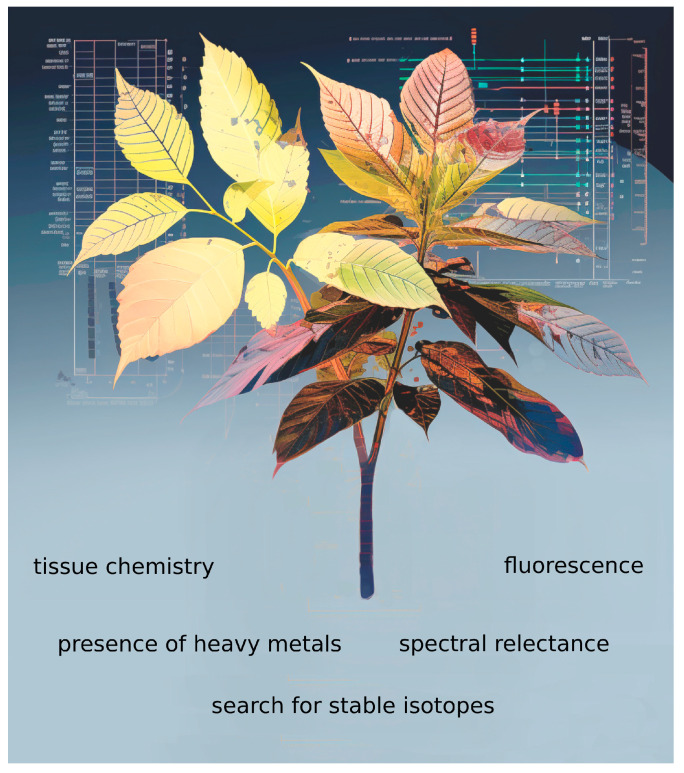
Different imaging techniques can be used for the study of herbarium collections (without damaging specimens) to collect data that can be integrated with morphological and genetic data.

The availability of large collections of herbarium specimens also favors analyses with imaging techniques involving newly developed methods for spectral analyses [19,20]. For instance, a reflectance spectrum across a range of wavelengths may be used to perform measurements of the chemical and structural composition of plants, whereas patterns in an ultraviolet spectrum, as perceived by pollinators, can be used as a diagnostic character to distinguish morphologically similar species [19,21]. At the same time, non-destructive specimen measurements of trace elements can be obtained using X-ray fluorescence, looking for trace metal hyperaccumulation. Interestingly, these multispectral analyses can be combined with tools based on artificial intelligence (AI) technology (Figure 2) enabling computer-automated (or semiautomated) measurement for large-scale analyses of phenological traits in thousands of specimen images [19,22,23].

Numerous best practices have been published in order to have standardized digital herbaria [14,15,16], whereas herbarium-derived measurements still represent a negligible minority of the available trait records. Similarly, a direct link between data and voucher specimens is not present, and specimen-derived data are not available as annotations or metadata for each studied herbarium sample, whereas data ideally should be permanently archived as newly obtained specimen records [19]. Although some data repositories, such as *MorphoBank* (https://morphobank.org/, accessed on 30 October 2023) and *Dryad* (https://datadryad.org/, accessed on 30 October 2023), are currently used by several researchers to publicly archive available data sets from individual studies, this approach may not allow for fast data retrieval that could be facilitated by directly recording traits and other phenological or chemical/physical data as metadata associated to each herbarium specimen.

Parallel with digitization, some international initiatives, such as the *Global Genome Biodiversity Network* (GGBN), have promoted and facilitated research projects connecting molecular analyses to voucher specimens in order to facilitate the verification of previous taxonomic determinations [24,25]. Similarly, the *Global Genome Initiative for Gardens* program (https://ggi.si.edu/ggi-gardens, accessed on 30 October 2023) is sampling and preserving at least one living species from each of the approx. 460 vascular plant families and one species from half of the approx. 15,000 vascular plant genera in order to build a collection of vascular plant biodiversity that can be used for research at the genome level. Similar initiatives are, therefore, playing a valuable role in current research on plant biodiversity also allowing for an improved understanding of plant evolution and adaptation to past, present, and future climate changes [10].

## 2. Herbaria and the DNA Sequencing Revolution

Natural history collections represent a unique source of information to understand habitat and biodiversity changes since they consist of temporally deep and spatially broad time series of samples [10]. Given their relevance to mapping biodiversity in different times and places, several natural museum collections have been studied at a molecular level, and numerous laboratories have analyzed the effects of different preservation methods on DNA [26,27,28,29].

In recent decades, hundreds of protocols have been used more or less satisfactorily for DNA extraction and sequencing from herbarium plant specimens, but these approaches are generally costly and require time-consuming optimization of the extraction protocols [26,27,28,29]. Molecular analyses of herbaria have been favored by the advent of next-generation sequencing (NGS) methods that allowed researchers to obtain molecular data, as well as whole-genome sequences, from old/ancient specimens even if they possess degraded DNA [10,30,31]. DNA from herbaria is generally classified as ancient DNA (aDNA) since it is usually degraded and fragmented, with fragments generally not exceeding 500 bp in length [32,33]. At the same time, cytosine-to-thymine misincorporations have also been observed in plant aDNA from herbarium collections, but post-mortem miscoding lesions represent a negligible source of errors in herbarium specimens [10,30,31].

The absence of adequate DNA integrity frequently makes herbarium specimens unsuitable for DNA Sanger sequencing, which usually requires long and intact DNA fragments, whereas NGS sequencing techniques are generally based (with the exception of third-generation NGS methods) on short sequences. Thus, these new technologies have enabled researchers to directly investigate plants conserved in herbaria in order to better understand numerous biological processes including, for instance, plant responses to anthropogenic global climate and environmental changes [32,33].

Even if several limitations are still present (for instance de novo assembly of the whole genome is very difficult using herbarium specimens), molecular analyses are quite common in herbarium collections, and different pipelines have also been satisfactorily applied in cases of very old specimens, such as vascular plants of about 200 years old and 100 years old lichens [32,33,34]. Regarding this, Papalini et al. [10] recently analyzed the available methods for handling DNA samples extracted from herbaria specimens, evidencing both strengths and limitations of the available pipelines. According to their analysis [10], even if both the quality and quantity of the extracted DNAs are lower in herbarium specimens than in fresh plant tissues, these samples are still suitable for amplification and NGS sequencing [10].

At the same time, the limited amount of DNA that can be extracted from herbarium specimens does not represent a limitation of current molecular analyses since it is possible to prepare high-quality NGS libraries using extremely low amounts of DNA, without prior sonication of the DNA samples from herbarium specimens, since they are already fragmented [35]. A low amount of DNA is particularly useful in museum natural collections since preserving the integrity of museum specimens, which prioritizes maintaining them in their original state, recurrently poses significant challenges for molecular analyses [10]. Furthermore, several authors introduced non-invasive approaches to sampling material directly from herbaria or methods that use a minimal amount of plant tissues [36,37]. These methods have also been successively applied to species with small (i.e., <25 mm^2^) and fragile leaves [10], thus enlarging the range of biological processes that can be studied at a molecular level using herbarium collections. Moreover, the cost-effective sequencing of herbarium specimens enabled a tremendous increase in the quantity of available molecular data that can be used for testing (phylo)genetic, demographic, and genetic hypotheses. As a whole, herbarium collections can make plant material available that is otherwise difficult to collect since it is related to rare or extinct species or is costly to obtain.

As recently reviewed by Burbano and Gutaker [38], herbarium collections can be used to trace the evolutionary history of wild plants and to better understand the effects of agriculture on plant biodiversity, the turnover of cultivated plant varieties over time, and the role of their hybridization with native species. Indeed, several cultivated plants have been moved from different countries so that herbaria represent a sort of time machine for the study of plant evolution at molecular and genomic levels [38]. In addition, the detrimental effects of land use change and habitat fragmentation can also be studied by integrating genomic and phenological data from herbarium specimens, which makes herbaria useful for reconstructing the profound influence of human activities on plant evolution [38]. Indeed, herbaria can make evident the extent to which the extinction and the arrival of invasive species reorganized plant communities in the Anthropocene in different habitats, as a consequence of intentional or unintentional plant relocations favored by humans in the last 500 years [39].

According to several published papers [10,38], molecular analyses will become the standard for herbarium collection-based genetic projects. This emphasis could be increased with DNA sequencing technologies developed by PacBio and Oxford Nanopore, which are not based on PCR amplification [40]. These approaches enabled cost-effective sequencing and allowed us to overcome some troubles related to the use of poor-quality aDNA. Indeed, several secondary compounds (including polyphenolics and polysaccharides) can covalently bind to DNA or co-precipitate with DNA, inhibiting PCR amplification even using non-degraded DNA samples. Interestingly, PacBio and Oxford Nanopore sequencing can overcome these troubles, thus making molecular analyses more common than expected [10,38].

Even if, at present, several characteristics have been identified as being predictive of successful DNA extraction and sequencing (including sample age, the specimen preservation method, storage climate, the method of drying, and leaf greenness), much of the literature pertaining to their identification is related to Sanger sequencing. Thus, the success of NGS studies based on aDNA samples from herbarium specimens is presently unpredictable. As a consequence, numerous challenges related to aDNA recovery and analysis (including the structure of the bioinformatic pipelines used for data analysis) have to be clearly identified in order to mitigate them in future NGS projects on herbarium samples [10]. As a whole, the inclusion of herbarium specimens in phylogenomic studies is potentially the most important remaining frontier for generating comprehensive global phylogenies needed to answer the most compelling long-standing questions in plant ecology and evolution [10].

These technical opportunities are the basis of the availability of molecular data obtained from herbarium samples in the recent decade [10,38], but a link between herbarium vouchers, DNA extracts, and molecular data is frequently absent. The curation of such collections and the maintenance of a dynamic link between them will provide a long-lasting and reliable framework for taxonomic investigations and will permit the critical re-evaluation of taxa delimitations at any time, based on both herbarium and DNA material. Indeed, herbarium vouchers may serve as physical evidence for the taxonomic identification of genome assemblies and could facilitate the correct identification of taxa or enrich the amount of data associated with each specimen. Interestingly, one of the main goals of the GGBN is the creation of infrastructure that can be used to encourage and enable scientists to complete the documentation chain connecting morphological vouchers, physical DNA samples, nucleic acid sequences, and publications. However, at present, no existing platform has efficiently aggregated biodiversity biobank data. 

## 3. Botanical Collections in Italy and the Italian National Biodiversity Future Center

Given their role in scientific research in the field of botanical systematics and phytogeography, herbarium collections are present in almost every state in the world. However, several nations often have numerous collections of great historical and scientific value [41].

Public institutions in Italy conserve 164 herbaria with high scientific, historical, and educational significance, often dating back to the seventeenth–nineteenth centuries, considering exclusively collections of dried plants (those once called horti sicci), whereas figurative herbaria or horti picti have been not considered [41,42]. The list of available herbaria includes 68 collections that are internationally recognized as parts of the *Index Herbariorum*, 63 herbaria not mentioned in the *Index Herbariorum*, but having a certain historical and scientific value (mostly belonging to museums, libraries, or scientific institutions of various types), and 33 collections deposited in various types of educational institutions, generally with mainly (or only) an educational value [41,42].

The Italian collections indexed in the *Index Herbariorum* belong to universities (n. 42), museums of local authorities or public scientific institutes (n. 20), religious institutes (n. 3), or other types of structures (n. 3). Among the oldest and scientifically important collections, a particular mention is related to the herbarium of the naturalist Ulisse Aldrovandi (1522–1605), containing many samples of plants from America (such as tomato, pepper, sunflower, pumpkin, prickly pear, etc.), known as the former specimens arrived in Europe [43,44,45].

Interestingly, the Italian botanical collections also include non-European plant specimens from Africa, as assessed, for instance, by the herbaria of Florence, Rome, Palermo, Turin, and Modena, which contain important collections from the 19th and 20th centuries, consisting mostly of plants from East Africa (Eritrea, Ethiopia, Somalia, Kenya), Northern Africa (Libya, Morocco, Algeria), and South Africa. Plants from South America are widely represented in numerous herbaria (Rome, Florence, Pisa, Pavia, Turin, etc.), especially from Brazil and the Pacific states (Ecuador, Peru, and Chile), whereas collections from Central America are less abundant. Numerous plant specimens from Asia are present in herbaria in Florence and Rome (essentially from Sumatra, New Guinea, and the Philippines), whereas plant collections from Oceania (Australia, New Zealand, Pacific islands) and Antarctica are scarcely present [41,42].

In Italy, 34 private herbaria are also present, and they consist, as a whole, of more than 156.000 specimens [45]. The richest private herbarium (20,000 samples) is the *Herbarium Antonietti* (conserved in Piedmont), followed by *Herbarium Soldano* (in Piedmont), possessing 18,100 samples, and the *Herbarium Branchetti* (in Emilia-Romagna), made of 13,000 samples. Most of them consist of modern collections: the oldest herbarium is *Herb. Hölzl Norbert*, which started in 1960 and is currently conserved in Bolzano (Italy), followed by *Erbario Soldano*, which started in 1973. The most recent are *Erbario Pascale* and *Herbarium Caetani*, which both started in 2014 [46].

The interest in Italian herbaria has been greatly hampered in the last two years by the foundation of the *National Biodiversity Future Center* (NBFC, www.nbfc.it/, accessed on 30 October 2023) aimed at conserving, restoring, monitoring, and enhancing Italian and Mediterranean biodiversity. The NBFC is one of the five national centers dedicated to frontier research, involving 48 institutions and companies throughout Italy, specifically involved in the study of biodiversity on land and water ecosystems based on key enabling technologies and environmental sustainability [47,48,49,50]. Interestingly, NBFC activities are also focused on the enhancement of Italian natural history museums with the digitization of natural collections and the development of national samples and data repositories on biodiversity. In particular, the activities of NBFC involve the large-scale digitization of the herbaria preserved in the *Herbarium Centrale Italicum* in Florence (Italy), aimed at publishing images and metadata according to international standards that guarantee interoperability and full accessibility of data across different platforms. The *Herbarium Centrale Italicum*, whose foundation is due to the botanist Filippo Parlatore (who was called to direct the herbarium in 1842 by the Grand Duke of Tuscany, Leopold II of Lorraine), is the most important Italian botanical collection, consisting of more than 4 million samples [51,52,53].

This large-scale digitization project will increase the accessibility to herbaria, and it will also allow for the organization of high-throughput approaches and workflows that could be successively applied to other relevant Italian herbarium collections. It will also provide a wealth of data that will be useful for scientific research, conservation projects, and education programs. As assessed by similar projects [54,55,56], high-throughput digitization systems for herbarium specimens mounted to sheets could provide benefits to projects related to conservation and land management, as well as to the general public. Indeed, digital herbaria will enable open access to biodiversity data as per the FAIR principles (ensuring data are Findable, Accessible, Interoperable, and Reusable) [57].

A further element of interest in the NBFC project on herbaria is related to the use of robust computer vision and machine learning workflows for examining herbarium images and extracting quantitative traits from specimens in place of manual or semi-automated methods. The latter processes tend to be labor-intensive and do not scale beyond a few dozen or a few hundred images. On the contrary, the choice of NBFC to adopt machine learning-based workflows will augment and expedite researchers’ ability to parse, measure, and review digital specimens at any project scale.

The NBFC project on *Herbarium Centrale Italicum* (which will be completed before the end of 2025) is part of the numerous national and international initiatives for digitizing natural history collections that are currently making available a vast amount of biodiversity data for researchers through aggregated portals, such as *Global Biodiversity Information Facility* (GBIF, https://www.gbif.org, accessed on 19 October 2023) and *iDigBio* (https://www.idigbio.org/, accessed on 19 October 2023).

## 4. Future Challenges and Conclusions

Herbaria are precious sources of data referring to plant biodiversity at very different scales (from international to regional ones). Indeed, herbarium collections are frequently focused on a regional or even local scale with few species sampled at different time periods, making them very useful for understanding which plant species disappeared and modified their distribution and/or abundance as a consequence, for instance, of climate changes [58]. For instance, herbarium specimens may be part of the toolkit of plant conservationists, enhancing our understanding of historical, current, and future trends related to rare plants [59].

At the same time, considering that plants may be prone to higher rates of attacks by pest insects as a consequence of climate change, herbaria have been useful in studying changes in habitats at a local scale as well as on a global one [60,61,62]. Moreover, climate changes have also been related to biological invasions, and geo-referenced data obtained from herbarium specimens can be used for biogeographic studies to assess the past extent of specific taxa [63], to depict current distributions [64], and to predict potential range shifts in a scenario of global change [8].

Lastly, herbaria can be considered unique repositories of phytogeographical data and the most accurate data sources to reconstruct events that occurred in the past. As a consequence, the role of herbaria has widened in recent years, from cataloging the diversity of life to documenting biodiversity changes and tracing spatiotemporal bioinvasion patterns [9,65,66]. Indeed, changes in plant communities are generally well-documented in herbarium collections, especially in the early stage of invasion [9]. 

The ongoing national projects aimed at the digitization of herbaria in different countries (such as Italy and Germany) will therefore make new data available that may be aggregated for the study of changes in plant biodiversity, with the chance of combining data obtained from different natural collections for studying ecosystem changes at very different scales [24,55,67]. In particular, digital data from *Herbarium Centrale Italicum* specimens will be available in a public repository that will strongly resemble *e-ReColNat* (https://www.recolnat.org/fr/, accessed on 30 October 2023), the platform that gathers digital images for all the natural history collections from France. A similar national repository (called *Integrated Digitized Biocollections*, https://www.idigbio.org/, accessed on 30 October 2023) is also present in the United States, where the National Science Foundation, through its *Advancing Digitization of Biological Collections* (ADBC) program, developed a strategic plan to digitize and mobilize images and metadata associated with all the biological research collections of the country [68]. These projects are at various stages of implementation and operation, but each of them has limitations, so the resulting infrastructures remain an innovative yet incomplete patchwork of distributed data and archival resources. Therefore, a future challenge for NBFC will be related to supporting collaborations among different institutions with common policies for data management and access [68].

Current studies on herbarium collections are good examples of the interest of taxonomists to eagerly incorporate technological advances into their work. However, the numerous ongoing projects on herbaria should also emphasize the relevance of the work of taxonomists not only to conserve them but to also allow similar projects in the future.

At the same time, the current interest in the study of herbaria could also favor collection-based research projects that build frameworks to support taxonomic research, together with education programs for a new generation of taxonomists who will be involved in the conservation and study of herbarium collections in the near future. Indeed, as recently reviewed by Löbl and colleagues [66], we recommend immediately reviving taxonomic research and teaching at universities at the tenured professor level to secure the education of the next generation of taxonomists. Interestingly, the activities of the Italian NBFC will involve not only the digitization of museum collections but also the training of young taxonomists together with new tenured positions for taxonomists at Italian Universities.

The availability of data related to herbarium collections may also favor the discussion of the importance of compiling taxonomic data into a single validated and accepted global list. Indeed, as recently discussed by Lien et al. [69], presently, there is no system for the systematic aggregation and maintenance of species names and current classifications into a globally accepted list to meet scientific and societal needs (related, for instance, to plant conservation and trade).

The creation of a governance system for species taxonomy requires the engagement of the community of taxonomists and digitized herbaria exactly favoring this kind of collaborative interaction so that they can allow the identification of governance standards that are essential to the creation and maintenance of such a global species list. At the same time, taxonomists involved in herbarium digitization are also using different repositories (such as GBIF, the Catalogue of Life, and ChecklistBank) so that they can also suggest what “entity” can fit the required standards for the maintenance of a global species list [69].

## Data Availability

No new data were created in this study.
